# Emerging paradigms in connectome-guided neurosurgery for central nervous system tumors: a narrative review of structural and functional connectomics

**DOI:** 10.1097/MS9.0000000000004915

**Published:** 2026-04-06

**Authors:** Tirath Patel, Hamza Yousuf Ibrahim, Muhammad Abbas, Muzna Amir Ali, Sana Sohrab, Kinza Nawaz, Syeda Farwa Zehra, Bhumi Daishik Patel, Nikhilesh Anand

**Affiliations:** a Department of Surgery, Trinity Medical Sciences University School of Medicine, Saint Vincent and the Grenadines; b Department of Surgery, Jinnah Medical and Dental College, Karachi, Pakistan; c Department of Surgery, Peshawar Medical College, Peshawar, Pakistan; d Department of Surgery, Dow University of Health Sciences, Karachi, Pakistan; e Department of Surgery, Azad Jammu Kashmir Medical College, Muzaffarabad, Pakistan; f Department of Surgery, Jinnah Sindh Medical University, Karachi, Pakistan; g Department of Surgery, Windsor University School of Medicine, Saint Kitts and Nevis; h Department of Medical Education, University of Texas Rio Grande Valley, Edinburg, TX, USA

**Keywords:** central nervous system tumors, connectomes, neurosurgery, surgical resection

## Abstract

Connectomics is a comprehensive mapping of structural and functional brain networks. It has introduced a paradigm shift in neurosurgery by redefining brain tumors as network disorders rather than isolated lesions. Unlike traditional surgeries, connectome-guided neurosurgery conceptualizes brain tumors as network disorders and aims to preserve brain function in resection. The objective was to study the role of connectome-guided neurosurgery in the management of Central Nervous System tumors and the use of structural and functional connectomics in tumor resection while preserving neurological function. Articles have been identified by searches of PubMed, Scopus and Google scholar from January 2000 to May 2025, using a combination of search terms like “connectomes,” “neurosurgery,” “central nervous system tumors,” “brain mapping,” “glioma surgery,” “tractography,” “neuroimaging,” “functional MRI,” “brain tumors,” “functional connectomics,” “structural connectomics,” “connectome-guided neurosurgery,” and “artificial intelligence” and addition of relevant references of articles. Included studies addressed both conventional and novel treatments for CNS tumors, including gliomas. Structural (DTI/tractography) and functional (fMRI, MEG, EEG) connectomes support preoperative planning, intraoperative guidance, and postoperative outcomes in CNS tumors. However, to reduce tumor size, a multiphase strategy is proposed by medical professionals, which includes rehabilitation, possible second surgery, and chemotherapy. Limitations of this study include tractography errors, fMRI neurovascular coupling, high costs, limited availability, and barriers to neurosurgeon training. Connectome-guided neurosurgery provides a paradigm shift from lesion-centric to network centric neurosurgery of CNS tumors. Although challenges remain, the use of connectomics with AI, intraoperative, and supramaximal resection strategies provides safe resection, reduces morbidity, and improves quality of life.

## Introduction

The connectome refers to the point-to-point mapping of neuronal circuits in the brain[1]. It is a complete representation of the brain’s structural (anatomical) and functional connections, conceptualized as interconnected networks[2]. The structural connectome is a complex network that explains anatomical connections between brain components[3], whereas the functional connectome maps the brain’s functional connections[4]. With the availability of both structural and functional connectomes, large-scale computational models of brain activity can be constructed[5]. The interaction between glial tumors and structural and functional neural networks is being increasingly recognized. It is redefining our understanding of the impact of these infiltrative lesions on global brain function[6]. Connectome analysis appeals to neurosurgeons not just because of the premise of mapping brain connectivity, but also because it allows for intuitive modeling of lesions and plasticity[7]. Consequently, neurosurgery has been at the forefront of the paradigm shift from a localizationist to a network-based approach to brain mapping[8].

HIGHLIGHTS

- Connectome-guided surgery redefines brain tumors as network disorders and prioritizes preserving functional brain networks during resection.
- DTI/tractography, fMRI, MEG, and EEG generate patient-specific maps that improve surgical planning, intraoperative navigation, and postoperative outcomes.
- Connectomic maps aid craniotomy design and subcortical navigation, while direct cortical stimulation remains the gold standard for functional validation.
- Connectome-informed strategies enable supramaximal safe resections, reduce neurological deficits, and improve quality of life without compromising oncological control.
- Despite obstacles such as cost, access, training gaps, and imaging limitations, advances in AI and real-time intraoperative mapping are expected to enhance precision and outcomes.

In this context, eloquent areas refer to the regions of the brain that are known to support specific functions, making people vulnerable to incapacity if these areas are damaged[9]. Neurosurgical planning, therefore, prioritizes avoiding damage to eloquent cortical and subcortical regions responsible for key functions, including movement, speech, language, and vision[10]. By providing a detailed map of individual patients’ brain networks, connectomic imaging enables the discovery of pathways that are essential but not traditionally eloquent for safe tumor resection[11]. Overall, connectome-guided neurosurgery provides a framework for understanding the complex relationships between tumors and neural networks, which guides surgical decisions and the development of strategies to preserve brain function. New approaches to this discipline combine enhanced imaging, computational modeling, and surgical mapping to improve patient outcomes.

## Connectomics in brain tumor surgery

Growing recognition of the connection between glial tumors and structural and functional neuronal networks is reshaping our understanding of how these infiltrative lesions affect brain function globally[6]. Connectomics, the science of mapping brain connectivity, has brought about a dramatic shift in the approach to glioma surgery[12], changing our view of brain tumors as network disorders rather than isolated masses[13]. With the advent of connectome imaging tools, anatomical and functional brain connectivity may now be visualized in patient-specific maps[10].

### Structural connectome

The structural connectome is a complex network of anatomical connections linking different brain regions[3]. Diffusion tensor imaging (DTI) of white matter allows the generation of a detailed map of structural neuronal connectivity on medium and large scales[14]. DTI is a non-invasive neuroimaging method for visualizing white matter pathways and predicting tumor locations[15]. It can provide significant diagnostic information to neurosurgeons[16].

Diffusion tensor imaging (DTI) and tractography are MRI techniques that can display white matter by tracing the diffusion of water molecules[17]. It has developed into a neurosurgical technique with applications in glioma surgery that are boosted by evolutions in crossing fiber visualization, edema correction, and automated tract identification[18].

There are certain limitations to DTI and tractography analyses. These techniques are user dependent and require a thorough understanding of neuroanatomy. Accurate interpretation also demands knowledge of fiber characteristics, including effects of brain shift, crossing fibers, and other relevant metrics[15]. Other difficulties that impair the interpretation and accuracy of DTI include false continuity, crossing fibers, fiber truncation, and edema[18].

### Functional connectome

A functional connectome is the mapping of the brain’s functional connections. It displays functionally diverse brain regions as network nodes and the connections between them as edges[4]. Nodes indicate brain areas, whereas edges represent statistical correlations of neuronal or BOLD activity between those regions[2]. Functional connectomics focuses on functional interactions within this circuitry, which are deduced from synchronized brain activity measured by functional MRI[19].

The fMRI is the most used noninvasive neuroimaging modality for surgical planning, and it has proven beneficial in tumor surgery[20]. Patients undergo fMRI scans while completing different motor activities in order to map motor function and uncover brain networks that are essential for motor performance[21]. Although fMRI is an effective method for researching how the brain works, it has drawbacks such as poor temporal resolution and sensitivity to patient movement[22].

Magnetoencephalography (MEG) is another neuroimaging technology that plays a crucial role in identifying brain illnesses. It detects magnetic fields created by intraneuronal currents, providing a direct index of neuronal activity and synaptic current[23]. It is closely related to electroencephalography (EEG), and ideally, both procedures should be interpreted simultaneously[24]. MEG provides a more direct evaluation of neuronal function than fMRI, which determines brain activity indirectly using blood-oxygen-level-dependent (BOLD) contrast[25]. Although MEG offers accurate brain mapping, its limitations include cost, technical complexity, and dipole sensitivity, which makes EEG more widely employed[26].

**Figure 1.**
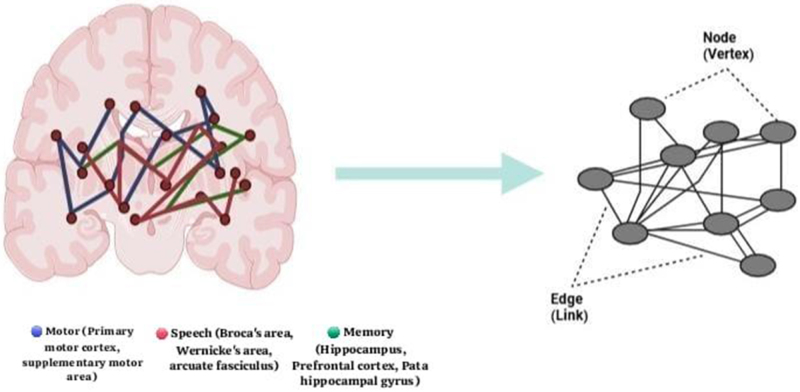
A conceptual diagram of the connectome that displays the nodes (brain areas) and edges (connections) that are part of the memory (green), speech (red), and motor (blue) networks. These are shown in the right panel as a simple graph with nodes (vertices) and edges (links).

Together, structural and functional connectomics give neurosurgeons patient-specific maps that improve surgical precision while conserving important networks. Despite some limitations, these techniques constitute a paradigm change in glioma surgery, reframing tumors as network illnesses rather than isolated lesions. Figure 1 provides a conceptual diagram of the connectome that displays the nodes and edges that are part of the memory, speech, and motor networks.

This manuscript is made compliant with the TITAN checklist to ensure transparency in the reporting of Artificial Intelligence[27].

### Methodology

This narrative review was conducted to comprehensively explore the emerging paradigms in connectome-guided neurosurgery for central nervous system tumors. The review sought to identify current use of connectomics in neurosurgery, ongoing research, and future directions in intraoperative connectome mapping and the integration of AI.

### Literature search strategy

A comprehensive literature search was conducted from January 2000 to May 2025 using PubMed, Scopus, and Google Scholar databases. Keywords used in the search were “connectome,” “neurosurgery,” “central nervous system tumors,” “brain mapping,” “glioma surgery,” “tractography,” “neuroimaging,” “functional MRI,” “brain tumors,” “functional connectomics,” “structural connectomics,” “connectome-guided neurosurgery,” and “artificial intelligence.”

### Inclusion and exclusion criteria

Peer-reviewed studies, clinical trials, meta-analyses, and potential review articles written in English were included. Studies emphasizing the structural and functional connectome mapping, emerging paradigms relevant to connectomics (e.g., AI-integration, real-time intraoperative connectome mapping, and connectome-based predictive modeling) were included. Conference abstracts, editorials, non-English studies, and animal studies were excluded.

### Data extraction and synthesis

Two reviewers independently screened titles, abstracts, and full-text assessments against the eligibility criteria. Any discrepancies were resolved through consensus or by consulting a third reviewer. The information extracted from each study included the study type, patient population, imaging or mapping technique used, surgical context (preoperative planning, intraoperative guidance, postoperative outcomes), and reported benefits or limitations. The data were thematically analyzed under key domains of structural connectome mapping (DTI/tractography), functional connectome mapping (fMRI, MEG, EEG), integration of multimodal connectomics, and intraoperative mapping innovations. Studies were critically appraised for methodological rigor, clinical applicability, and relevance to emerging paradigms in CNS tumor neurosurgery.

## Applications in glioma and other CNS tumors

A new understanding of tumor-brain interactions could be provided by the incorporation of connectomics into neuro-oncological paradigms, which has the potential to transform personalized medicine and treatment approaches[28]. Beyond the conventional localizationist perspective, this

The paradigm shift acknowledges that neurological processes originate from large-scale, interconnected brain networks rather than discrete areas. In order to maximize tumor excision while maintaining vital neurological functions, surgeons have an unparalleled chance to improve surgical techniques thanks to our growing understanding of the topology of the brain network and how neoplastic processes disturb it. Through the use of sophisticated imaging and computational methods, this method, known as connectome-guided neurosurgery, maps the neural networks unique to each patient, reducing functional deficits following surgery and enhancing quality of life[29].

### Preoperative planning using connectome maps

To generate patient-specific maps of cortical hubs and subcortical white-matter pathways that are vulnerable to resection, preoperative connectome mapping combines structural diffusion MRI tractography with functional evaluations (task-fMRI, resting-state fMRI, MEG, and nTMS). This multimodal method is specifically put out as a paradigm shift in glioma surgery, moving away from a lesion-centric strategy and toward a network-centric (hodotopic) strategy that takes distributed processing and plasticity into consideration[30].

Clinical teams report that in a significant subset of patients, connectome-informed planning changes the operative strategy (craniotomy site, cortical entry point, and resection corridor). This is especially true for tumors in or near perisylvian and insular regions, where language and association tracts are very vulnerable. These changes are explained in several articles and series that provide an overview of how nTMS and preoperative tractography improve surgical decision

making[31]. Major preoperative mapping cautions include heterogeneity in tractography techniques (deterministic versus probabilistic), differences in MRI acquisition parameters and processing pipelines, and tumor-related distortion or white matter infiltration that complicates automated reconstructions. The direct interchangeability of maps across centers has been restricted by these sources of variation[32].

### Intraoperative use (navigated TMS, tractography overlays)

To provide the surgeon with a continuous visual roadmap throughout resection, preoperative functional maps and tractography can be converted to neuronavigation devices and shown as intraoperative overlays. The nTMS offers non-invasive cortical mapping that can be used to seed tractography (nTMS-guided DTI) and registered to navigation, enhancing the anatomical plausibility of reconstructed tracts and assisting in the preoperative localization of motor and language cortices[33].

Although the evidence is still mostly observational and single-center, clinical series indicate that the combined intraoperative use of tractography overlays and nTMS-derived maps may improve the safety of resection by permitting more extensive tumor removal without increasing permanent morbidity in certain cohorts. Many centers combine overlays with intraoperative validation (awake mapping, direct cortical/subcortical stimulation, or intraoperative imaging) because brain displacement during resection gradually decreases overlay accuracy[34].

To maintain the usefulness of preoperative connectome information throughout the procedure, several groups have proposed dynamic or “intraoperative” white-matter navigation procedures (such as intraoperative MRI or ultrasound to account for brain shift). These methods have potential, but they need certain infrastructure[35].

### Case examples: insular gliomas, language-dominant hemisphere tumors

Insular gliomas are a classic example of connectomic mapping being useful because the insula is located next to associative and language tracts (such as the long segment of the arcuate fasciculus and extreme capsule fibers), and preoperative tractography often identifies “no-go” subcortical regions that affect the resection corridor and cortical entry. According to several case series, planning based on functional mapping and tractography lessens early postoperative language impairments without necessarily sacrificing the extent of resection[34]. While not flawless, a number of comparative investigations have demonstrated a respectable concordance between nTMS and intraoperative direct cortical stimulation for language mapping. Direct stimulation is still the gold standard, although nTMS is a helpful preoperative supplement because alert intraoperative testing may be limited by fatigue and length[36].

## Integration with awake mapping

### Connectome as a guide versus direct cortical stimulation

During awake craniotomy, direct electrocortical stimulation (DES/DCS) provides real-time physiological confirmation of eloquent cortex and remains the gold standard for mapping movement and language functions during surgery. Rather than serving as a substitute for stimulation, preoperative connectome maps are best described as tools that assist in identifying and prioritizing areas for DES because they are intrinsically probabilistic and offer a roadmap rather than precise functional localization[37].

### Complementary versus competing approaches

The majority of the literature presents awake mapping and connectomic approaches as complementary. While awake mapping provides the physiological confirmation required for safe resection, connectome maps help with craniotomy planning, reduce the search space for stimulation, and offer subcortical tract information that DES cannot directly observe. According to comparative assessments, nTMS and DES have a respectable correlation for motor mapping and exhibit encouraging but inconsistent findings for linguistic mapping; as a result, nTMS/connectome techniques are helpful supplements but not perfect replacements[35]. To estimate functional boundaries in patients who are unable to undergo awake procedures, doctors may rely more on connectome-based workflows (nTMS + tractography + rs-fMRI); nevertheless, these connectome-only methods are not as well validated and should be used with caution. There remains a need for prospective comparative studies that compare the functional and oncologic results of integrated processes to DES-only methods[38].

Table 1 summarizes key studies evaluating connectomics-based techniques in neurosurgical oncology, highlighting their clinical impact on mapping motor, language, and cognitive networks.

**Table 1:** Summary of studies applying connectomics in neurosurgical oncology.

Study	Network targeted	Technique	Clinical impact
Jeltema, *et al*[34]	Motor & Language Networks	Systematic Review & Meta-Analysis of nTMS vs DCS	Provides a comprehensive overview of the literature, concluding that nTMS is a valuable preoperative adjunct for motor mapping (accuracy 2–16 mm vs DCS) and a useful but more variable technique for language mapping (high NPV), but it cannot take the place of intraoperative DCS mapping.
Lakhani, *et al*^[^35^]^	Sensorimotor, Language, Visual	Task-based fMRI, Resting-state fMRI	fMRI aids in mapping but requires DCS because it is unreliable on its own, particularly for language; there is still little proof that it has an independent outcome benefit.
Qiu, *et al*[36]	Motor Network	Intraoperative Resting-State fMRI (iR-fMRI)	Demonstrated feasibility of real-time motor mapping during surgery. Achieved 61.7% sensitivity and 93.7% specificity vs DCS. Filling the tumor cavity with saline significantly improved image quality.
Samuel, *et al*[33]	Multiple Functional Networks (e.g., Cognitive, Language, Motor)	DTI, fMRI, MEG, EEG, ECoG, Awake Mapping	Examine the proposal for a network-based paradigm for glioma surgery. Claims that this strategy can preserve cognitive function, maximize “oncologic disconnection,” and direct neuromodulation for neurorehabilitation to enhance results
Sollmann, *et al*[37]	Motor & Language tracts	nTMS-based DTI tracking	Evaluation of surgical risk. Permanent deficiencies are predicted by the distance between the tumor and the nerve tract (e.g., ≤ 12 mm for motor, ≤ 16 mm for language.
Wei, *et al*[38]	Whole-brain structural network (connectome)	dMRI connectomics & graph theory	Measuring unseen tumor infiltration in normal-looking brain tissue makes an exact prognosis possible. Acts as a biomarker for imaging that more precisely forecasts survival and cognitive decline than conventional clinical assessments.

## Clinical outcomes

### Evidence of reduced deficits

Utilizing connectome gradient mapping, a recognized dimensionality reduction technique, to examine local-global brain interactions in individuals with pharmacoresistant temporal lobe epilepsy (TLE) and in patients with diffuse low-grade gliomas DLGGs is of utmost importance pre- and postoperatively, showing improvement in reduced neurological deficits and better quality of life[39]. Insights derived from connectomic and neuroimaging investigations of brain tumors possess considerable clinical significance. In neurosurgical planning, the objective has shifted from merely optimizing tumor excision to enhancing tumor removal while safeguarding functional networks, a notion frequently referred to as connectome-aware surgery. Recent years have proven that awake mapping utilizing cortical and axonal direct electrical stimulation, alongside intraoperative cognitive monitoring, significantly diminishes postoperative morbidity[40].

**Figure 2.**
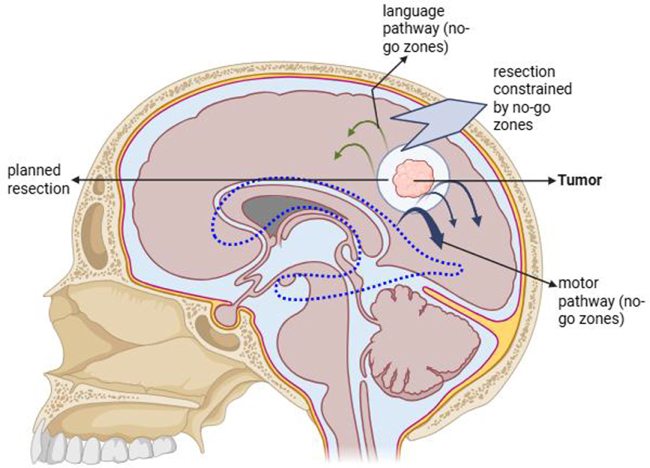
Schematic patient example: tumor resection constrained by connectome-informed no-go zones.

In addition to treating the tumor, the goal is to preserve and repair the networks in the brain in order to increase patient survival and quality of life. Neuroimaging scientists, neurosurgeons, neuro-oncologists, radiologists, and rehabilitation specialists will continue to improve these network-aware techniques through interdisciplinary collaboration. The best treatment plan for brain tumors is expected to look something like maximal safe excision with connectomic mapping in the upcoming years[13]. Figure 2 presents a schematic patient example illustrating how connectome-derived “no-go zones” constrain the extent and trajectory of tumor resection during surgery.

### Oncological outcomes versus functional preservation

According to Magnani *et al*, if we talk about the oncological outcomes, the connectome-based approach suggests that the surgical procedure might be modified to preserve the fibers essential for maintaining a satisfactory level of cognitive function while sacrificing those required to provide an effective EOR[41]. Early functionally guided surgery significantly improves DLGG patients’ functional and oncological outcomes. As written by Duffau *et al* To remove brain tumors such as diffuse low-grade gliomas (DLGG), doctors employ “awake mapping.” During surgery, this method keeps the patient awake and stimulates key brain regions to maintain vital processes. It enhances quality of life, lowers the danger of brain injury, and makes complete tumor removal possible. However, medical professionals may employ a multi-phase strategy that includes rehabilitation, possible second surgery, and chemotherapy to reduce the tumor’s size. Removing the tumor while maintaining quality of life is the aim[42].

## Challenges and limitations

Although connectome-guided neurosurgery has the potential for maximal tumor resection while preserving neurological function, several challenges must be addressed before routine clinical integration is feasible.

### Technical variability

Although Tractography and fMRI are two of the most essential tools currently used in connectome mapping, some issues must be resolved before they can become completely reliable. Tractography algorithms often produce ambiguous results and can generate false-positive fiber bundles in bottleneck areas, despite having high angular resolution diffusion imaging of the diffusion signal[42]. This can increase the risk of postoperative deficits triggered by the misidentification of the white matter tracts during surgical planning.

Functional MRI (fMRI) continues to be a valuable non-invasive preoperative functional mapping tool in patients with low-grade tumors. However, the reliability of fMRI is reduced in high-grade gliomas due to factors such as neurovascular uncoupling and impaired task performance. These conditions compromise the blood-oxygen-level-dependent (BOLD) signal, leading to both false-negative and false-positive activations. Therefore, fMRI should be used with other techniques to minimize these limits in patients with high-grade disease[43].

### Cost and availability

Connectome-guided surgeries require significant capital investments due to the use of imaging techniques and intraoperative integration platforms (e.g., iMRI, advanced neuronavigation). For instance, neuronavigation systems cost around USD 60 000, making them inaccessible to low-and middle-income countries (LMIC), where neuro-oncology infrastructure itself is limited^[^44,45^]^. Subsequently, many centers in LMICs rely on inexpensive alternatives, such as intraoperative ultrasound or modified surgical plans, to negate the need for advanced platforms. Additionally, a major hindrance in the broader implementation of connectome-guided neurosurgery is the unavailability of these neurosurgical platforms and the infrastructure and trained practitioners required to operate them[46].

### Training barriers for neurosurgeons

Although the data show the efficacy of connectome-guided neurosurgery, the lack of structured training during residency is a significant hurdle in its widespread adoption. Most training programs currently lack the training required to become fluent in advanced neuroimaging techniques, such as tractography interpretation and associated software tools. Furthermore, access to educational tools such as high-fidelity simulation, augmented reality modules, and expert subspecialty mentorship is lacking, especially in LMICs, resulting in a steep learning curve for health professionals[47].

## Future directions

Connectome-guided neurosurgery for CNS tumors is set for significant advancements by integrating revolutionary technologies and surgical strategies. Significant advances have been made in connectomics research in recent years as a result of the integration of AI. AI-powered predictive models can be developed to categorize neurological diseases and link functional activities with post-surgical outcomes and connectome pattern alternations.

Machine learning and deep learning are great tools for early diagnosis and prognosis of many neurological diseases. Deep learning algorithms called Block Decomposition and Stitching (BDS) have been shown to increase connectome mapping accuracy when combined with typical connectome pipelines by 20–30%, making it more accurate than conventional tractography alone. Thus, paving the way for automated, highly accurate connectome reconstructions that can enhance surgeons’ understanding of patient-specific architecture before and during surgery, leading to better post-surgical outcomes[48].

Alongside these developments, real-time intraoperative connectome mapping technologies, such as electrocorticography (ECoG), have emerged as critical tools in functional neurosurgery. ECoG allows surgeons to visualize functional brain networks during tumor resection procedures. For instance, Noll *et al* demonstrated that intraoperative ECoG with gamma band modulation leads to safe resection margins during awake craniotomy by providing immediate cortical heat maps of language function that closely correspond with direct cortical stimulation (DCS), which can decrease the risk of postoperative deficits[49]. Similarly, another study conducted by Erez *et al* reported that intraoperative ECoG could capture high-gamma power change attributed to executive function tasks, expanding the scope of functional mapping beyond motor and language domains[50]. These methods can lead to tumor resections that maximize tumor removal while also minimizing risks to critical brain functions^[^49,50^]^.

Supramaximal resection strategies integrated with connectomics are promising for optimizing oncological and cognitive outcomes. Preoperative and intraoperative connectome analysis guide safe resections by identifying critical pathways that maintain cognitive and motor functions. A correlation has been seen between neurocognitive outcomes and connectome disruptions, highlighting the prognostic utility of connectomes[51]. Surgeons can maximize tumor resection beyond the conventional margins while minimizing the risk of neurological deficits by combining resting-state fMRI, diffusion imaging, and AI-driven mapping tools to create connectome maps personalized to individuals[43].

## Conclusion

The management of CNS tumors like gliomas is evolving with the use of novel therapeutic strategies like connectome-guided neurosurgery. Besides personalized medicine and traditional treatment approaches, their limitations, particularly in functional loss, lack of precision, recurrence, and variable patient response, highlight the need for new treatment strategies. The transformative shift of connectome-guided neurosurgery acknowledges neurological processes as interconnected brain networks rather than discrete areas. Structural and functional connectomics preserve neurological functions when used by neurosurgeons during tumor resection. Advances in preoperative tractography, fMRI, BDS, and ECoG have enabled the creation of patient-centered brain network maps. Intraoperative integration with awake mapping and AI modalities further enhances surgical precision.

Despite its promising results, there are limitations like tractography errors, fMRI neurovascular coupling, high costs, low availability, and barriers of training to neurosurgeons, but this field is rapidly evolving with the use of AI-driven predictive models, awake mapping, block decomposition and stitching, real-time intraoperative connectome mapping, direct electrocortical stimulation, and supramaximal resection strategies. Collaborative, multidisciplinary efforts and standardized protocols combined with continued research could enhance the effectiveness of CNS tumor management and improve the quality of life. Finally, a network-based connectome-guided neurosurgery provides neurosurgical care with a balance of oncological control and preservation of cognitive and functional outcomes.

## Data Availability

All data used in this narrative review are publicly available and sourced from previously published studies. No new data were generated for this work. All included articles have been appropriately cited within the manuscript and are available through the references section.
